# Local delivery of doxorubicin prodrug via lipid nanocapsule–based hydrogel for the treatment of glioblastoma

**DOI:** 10.1007/s13346-023-01456-y

**Published:** 2023-10-27

**Authors:** Mingchao Wang, Raphaël Bergès, Alessio Malfanti, Véronique Préat, Chiara Bastiancich

**Affiliations:** 1 UCLouvain, Louvain Drug Research Institute, Advanced Drug Delivery and Biomaterials, Avenue Mounier 73, 1200 Brussels, Belgium; 2 Aix-Marseille University, CNRS, INP, Inst Neurophysiopathol, 27 Boulevard Jean Moulin, Marseille, 13005 France; 3 Department of Drug Science and Technology, University of Turin, Via Pietro Giuria 9, Turin, 10125 Italy

**Keywords:** Glioblastoma, Local delivery, Lipid nanocapsules, Hydrogel, Doxorubicin

## Abstract

Glioblastoma (GBM) recurrences appear in most cases around the resection cavity borders and arise from residual GBM cells that cannot be removed by surgery. Here, we propose a novel treatment that combines the advantages of nanomedicine and local drug delivery to target these infiltrating GBM cells. We developed an injectable lipid nanocapsule (LNC)–based formulation loaded with lauroyl-doxorubicin prodrug (DOXC_12_). Firstly, we demonstrated the efficacy of intratumoral administration of DOXC_12_ in GL261 GBM-bearing mice, which extended mouse survival. Then, we formulated an injectable hydrogel by mixing the appropriate amount of prodrug with the lipophilic components of LNC. We optimized the hydrogel by incorporating cytidine-C_16_ (CytC_16_) to achieve a mechanical stiffness adapted for an application in the brain post-surgery (DOXC_12_-LNC^CL^). DOXC_12_-LNC^CL^ exhibited high DOXC_12_ encapsulation efficiency (95%) and a size of approximately 60 nm with sustained drug release for over 1 month in vitro. DOXC_12_-LNC^CL^ exhibited enhanced cytotoxicity compared to free DOXC_12_ (IC_50_ of 349 and 86 nM, respectively) on GL261 GBM cells and prevented the growth of GL261 spheroids cultured on organotypic brain slices. In vivo, post-surgical treatment with DOXC_12_-LNC^CL^ significantly improved the survival of GL261-bearing mice. The combination of this local treatment with the systemic administration of anti-inflammatory drug ibuprofen further delayed the onset of recurrences. In conclusion, our study presents a promising therapeutic approach for the treatment of GBM. By targeting residual GBM cells and reducing the inflammation post-surgery, we present a new strategy to delay the onset of recurrences in the gap period between surgery and standard of care therapy.

**Graphical Abstract:**

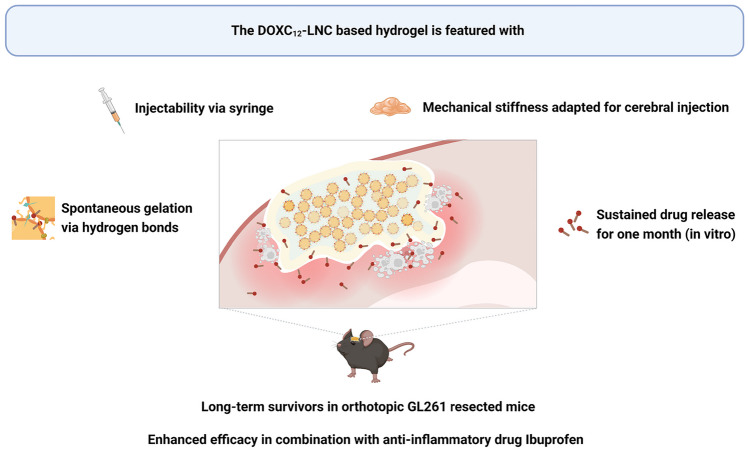

## Introduction

Glioblastoma (GBM) is an aggressive and fatal primary brain tumor that mainly affects adults. Despite the standard of care treatment—which includes surgery followed, several weeks later, by oral temozolomide (TMZ) chemotherapy and radiotherapy—GBM patients face a dismal prognosis [1]. Safe surgical debulking of the tumor does not always allow the removal of all GBM cells that present an infiltrative behavior into the brain parenchyma. Consequently, the recurrence of aggressive and chemoresistant tumors occurs in virtually all patients, and 90% appear in proximity of the post-surgical margins [2]. Achieving efficient drug delivery to brain tumors is still a challenging task due to the presence of various biological barriers and delivery challenges.

The blood–brain barrier (BBB) presents a significant obstacle to brain drug delivery, restricting the access of drugs to central nervous system (CNS) [3]. Comprised of specialized endothelial cells connected by tight junctions and supported by interactions with basement membranes, brain pericytes, astrocytes, and neurons, the BBB safeguards the brain by preventing the passage of macromolecules, pathogens, and neurotoxins. Only small (< 500 Da), lipid-soluble molecules can easily traverse the BBB and reach the CNS, leaving 90% of small-molecule and larger therapeutic drugs blocked in the bloodstream [4, 5]. Although the BBB’s permeability may be compromised by the tumor, systemically administered molecules struggle to reach individual infiltrating tumoral cells where the BBB remains less altered in GBM patients [6, 7]. To surmount this challenge and achieve effective drug delivery across the BBB, various strategies have been explored, including physically disrupting the BBB, employing chemical modifications with prodrugs and nanocarriers, and implementing interstitial delivery to bypass the BBB [8].

Chemotherapy resistance creates another significant challenge for the clinical management of GBM. Alkylating agent temozolomide is included in the current standard of care treatment of newly diagnosed GBM patients [9]. It is administered orally as it can cross the BBB and convert spontaneously into its active metabolite, methyltriazeno-imidazole-carboximide (MTIC), through hydrolysis under physiological conditions [10]. This active form delivers a methyl group to purine bases of DNA, leading to unrepairable mismatches and cellular apoptosis [11]. However, less than half of GBM patients respond to alkylating agent treatment, with some patients exhibiting innate or acquired chemoresistance [12]. One of the mechanisms of the chemoresistance is mediated by the DNA repair enzyme O_6_-methylguanine methyltransferase (MGMT) which can eliminate the cytotoxic O6-methylguanine DNA adduct before it causes harm. For combinatory approaches, it is recommended to use drugs with a different mechanism of action to avoid cross-resistance with alkylating agents [13].

Local drug delivery at the tumor site offers a promising strategy for GBM treatment bypassing the BBB and achieving a therapeutic concentration while minimizing systemic side effects [13]. Moreover, using drug-loaded scaffolds to be administered in the post-surgical cavity can ensure sustained drug release in the gap time between surgery and standard of care GBM therapy. Carmustine-loaded wafer Gliadel^®^ is approved for GBM patients [14], but it is not included in the European Association of Neuro-Oncology guidelines for the treatment of GBM [9]. Indeed, its rigid structure, low adherence to the resection cavity borders, fast drug release, and the appearance of local side effects limit its use in the clinical practice [15, 16]. Therefore, safer and more effective local treatments adapted to the post-surgical cavity and able to guarantee a sustained release of active drugs are desired to exploit the potential of this route of administration.

Hydrogels hold promise as a local drug delivery system due to their injectability and soft composition, making them ideal for post-surgical implantation in the brain. They can adapt to the irregular shapes of the tumor resection cavity, overcoming the limitations of rigid wafer implants and offering controlled drug release through compositional adjustments or mechanical strength modifications by using bioadhesive polymers [17]. Recently, the emerging field of nanomedicine-based hydrogels has gained attention for its combined benefits of nanomedicine and local delivery [18]. Nanocarriers are employed to improve drug solubility, protect drugs from degradation, enable sustained drug release, and selectively target specific cell populations through surface coating or modification [19]. Several nanomedicine-based local treatments employing hydrogel matrices have been developed for GBM treatment [20–22]. Among these, lipid nanocapsule (LNC)–based hydrogels exhibit great potential for anti-GBM therapy. Composed of an oily core and an amphiphilic surfactant shell, LNCs can spontaneously form hydrogels when incorporating amphiphilic molecules, such as lauroyl-gemcitabine (GemC_12_), into their formulation [23, 24]. Alternatively, palmitoyl-cytidine (CytC_16_) has been utilized as a biocompatible cross-linker to form LNC-based hydrogels without altering the size distribution of LNCs [24]. In these LNC-based hydrogels, the hydrophilic moieties of the amphiphilic molecules on the surface of the nanocarrier form a hydrogel through H-bond interactions, while hydrophobic chains are entrapped in the oil–water interface of the LNCs. Despite a demonstrated safety and anti-cancer efficacy in several GBM models, GemC_12_-LNC hydrogels have not led to an inhibition of tumor recurrence in the long term [23, 25, 26]. Therefore, it is necessary to develop more potent drug delivery systems while exploiting this technology. In this study, we aim to develop a new LNC-based hydrogel by modifying the active drug to enhance the anti-GBM potency. To achieve this, we will replace GemC_12_ with a doxorubicin (DOX) derivative prodrug.

Doxorubicin is an effective chemotherapy drug used to treat cancer alone or in combination with other drugs [27]. It is approved by the FDA to treat breast cancer, bladder cancer, Kaposi’s sarcoma, lymphoma, and acute lymphocytic leukemia, among others. Although DOX has not yet received approval for GBM treatment, it has been tested on GBM patients in several clinical trials (NCT02758366; NCT01851733). Preclinical studies have shown DOX effectiveness against multiple GBM cell lines, including human U251 GBM cells, U87-MG cells, T98G GBM cells, and murine GL261 GBM cells [28, 29]. In vivo, both systemic administration and local delivery of DOX through various techniques have been reported to inhibit tumor growth in orthotopic GBM animal models [30, 31]. DOX primarily kills cancer cells by intercalating into their DNA, disrupting topoisomerase-II-mediated DNA repair, and damaging cellular membranes, DNA, and proteins through increased free radicals [32]. Importantly, DOX operates through a different mechanism compared to TMZ, thus avoiding the extensively reported resistance mediated by alkylating agents [33]. DOX is commercialized as water-soluble doxorubicin hydrochloride but for successful incorporation into an LNC-based hydrogel, a DOX prodrug with increased amphiphilicity is required. In previous work, we successfully modified DOX by creating a lauroyl hydrazone derivative (DOXC_12_) [34]. DOXC_12_ represents a promising DOX prodrug candidate preserving similar IC_50_ as DOX in murine GL261 cells and human U87-MG cells, along with faster cellular uptake due to the lipophilic C_12_ lauroyl chain [34]. Additionally, the pH-cleavable hydrazone linkers in DOXC_12_ can lead to controlled DOX release in an acidic environment. Besides, the presence of an aliphatic chain should promote the presence of DOX at the oil–water interphase of LNCs and allow the formation of H-bonds between LNCs thus forming a hydrogel [24].

Inflammation is a critical component of tumorigenesis and progression for various forms of cancer [35]. In response to a brain lesion in a normal tissue, processes like inflammation, tissue replacement, and remodeling are initiated as part of the healing process, and they stop over time. However, the acute inflammation induced by surgical brain injury does not resolve due to the presence of residual cancer cells at the resection cavity borders, leading to a chronic inflammatory protumorigenic microenvironment which can boost the onset of recurrences [36]. Ibuprofen, classified as one of the non-steroidal anti-inflammatory drugs (NSAIDs), has been documented to effectively eliminate micrometastases in various tumor-resection models, ultimately enhancing survival rates through its early suppression of the inflammatory cascade [37]. We hypothesize that the administration of an anti-inflammatory drug following surgery might control the post-surgical inflammation. Its combination with the local chemotherapeutic DOXC_12_-LNC treatment could modulate the post-surgical microenvironment to prevent the onset of recurrences.

**Fig. 1:**
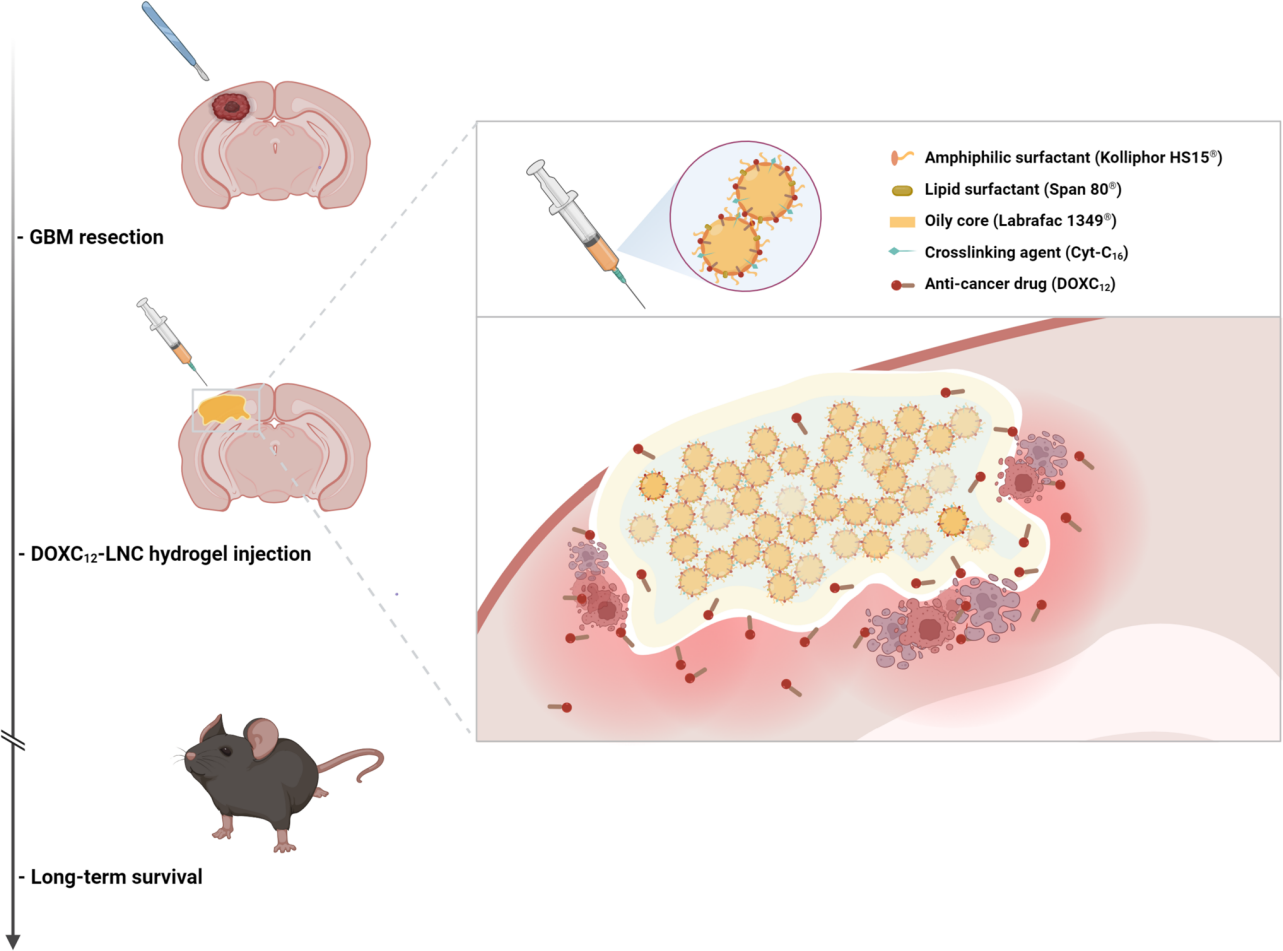
Schematic illustration of DOXC_12_-LNC^CL^ hydrogel for preventing post-operative GBM relapse. The injectable DOXC_12_-LNC^CL^ hydrogel is formulated to conform to the irregular GBM-resected cavity. CytC_16_ was incorporated to obtain adapted mechanical properties for cerebral administration. Upon degradation, DOXC_12_ is released from the DOXC_12_-LNC^CL^ hydrogel and diffuses into the surrounding brain tissue. This local delivery allows DOXC_12_ to reach and kill residual infiltrating GBM cells at the resected margin. Consequently, the onset of GBM recurrence is delayed and long-term survival is achieved

In this study, we aimed to develop a nanomedicine-based local treatment utilizing DOXC_12_ and LNC to eliminate residual infiltrating GBM cells in the tumor resection cavity margins, thereby preventing tumor recurrence. To achieve a sustained anti-cancer effect, we designed an LNC-based hydrogel incorporating the amphiphilic DOXC_12_ (Fig. 1). Initially, we assessed the anti-cancer efficacy of DOXC_12_ through intratumoral administration in GBM-bearing mice. Subsequently, we formulated a DOXC_12_-LNC hydrogel, and to optimize its rheological properties, we introduced CytC_16_, achieving the appropriate DOX content in the hydrogel (DOXC_12_-LNC^CL^). The hydrogel was extensively characterized, and its anti-cancer efficacy was evaluated in various preclinical models. Finally, to seek an enhanced therapeutic effect, we combined the local treatment of DOXC_12_-LNC^CL^ with the parenteral administration of the anti-inflammatory drug ibuprofen.

## Material and methods

### Synthesis of doxorubicin lauroyl hydrazone derivative (DOXC_12_)

The synthesis of DOXC_12_ was carried out with the method previously reported [34]. Briefly, 100 mg of doxorubicin hydrochloride (Chemieliva, China) and 53.5 mg of dodecanoic hydrazide (ChemCruz, Netherlands) were dissolved in methanol. Further, the solution was mixed with 100 µL of glacial acetic acid (Merck, USA) in a round-bottomed flask at 40 ℃ overnight. The product was precipitated by adding diethyl ether (Avantor, USA) and was collected by centrifugation at 4 °C. The final precipitate was freeze-dried (Labconco, USA) and analyzed by ^1^H NMR and HPLC analysis before use as previously reported (data not shown) [34].

### Formulation of DOXC_12_-LNC hydrogel

To formulate DOXC_12_-LNC, we adapted the phase-inversion technique method previously reported [23]. Briefly, 438 mg of Labrafac^®^ 1349 (Gattefosse, France), 40 mg of Span 80 (Sigma-Aldrich, USA), and 412 mg of Kolliphor^®^ HS15 (Sigma-Aldrich, Germany) were mixed in a vial with stirring and mild heating for 1 h. Once a homogeneous suspension was obtained, 40 mg of DOXC_12_ was added to the system in a water bath at 50 °C until the complete dissolution of the drug. Then, 15 mg of sodium chloride (VWR Chemicals, Belgium) and 350 mg of ultrapure water were added to the formulation at room temperature. Three cycles of heating and cooling were performed between 45 and 75 °C under magnetic stirring (500 rpm). At the phase-inversion temperature in the last cooling cycle, 700 mg of ultrapure water was added, and the formulation was stirred for 1 more minute. Before gelation, DOXC_12_-LNC solution was inserted in insulin syringes (BD Micro-Fine™ needle; Becton Dickinson, France) and stored at 4 °C until further use. Theoretical drug loading of DOXC_12_ within the DOXC_12_-LNC hydrogel was 9% (w/w), corresponding to 7% (w/w) of DOX equiv. The blank LNC was obtained using the same method without adding DOXC_12_.

A formulation of DOXC_12_-LNC and blank LNC containing 4-N palmitoyl-cytidine (CytC_16_) (Atlanchim Pharma, France) was also prepared using the same procedure, and 15 mg of CytC_16_ (3.4%, w/w) was added in the vial before the heat-cooling cycles as previously reported [24]. The systems containing CytC_16_ will be referred to as DOXC_12_-LNC^CL^ and blank LNC^CL^.

### Physicochemical characterization

#### Size, zeta potential, and drug loading capacity

The average size and polydispersity index (PDI) of formulations were measured by dynamic light scattering, and zeta potential was measured by laser Doppler velocimetry using apparatus Zetasizer NanoZS (Malvern Instruments, UK) equipped with a red laser (*λ* = 633 nm) at a fixed angle of 173° at 25 °C. Measurements of samples were performed in 60 times dilution ultrapure water (*n* = 3).

#### Short-term stability of DOXC_12_-LNC^CL^ hydrogel

The physical stability of the DOXC_12_-LNC^CL^ and blank LNC^CL^ was evaluated by measuring the average size, PDI, and zeta potential after storage for 2 weeks at 4 °C (*n* = 3).

#### Quantitative determinations of DOXC_12_ content in the hydrogel

The free DOXC_12_ in the DOXC_12_-LNC^CL^ hydrogel was collected by ultrafiltration. Briefly, DOXC_12_-LNC^CL^ hydrogel was diluted with water. The DOXC_12_-LNC^CL^ solution was centrifugated at 10,000 g for 15 min using a Vivaspin 500 filter (10 kDa; Cytiva, USA). The bottom aqueous solution isolated from the filter was labeled as free DOXC_12_. In parallel, a DOXC_12_-LNC^CL^ solution without centrifugation was diluted by methanol (1:20) and labeled as total DOXC_12_.

The amount of DOXC_12_ in the LNC was quantified with high-performance liquid chromatography (HPLC), using a Shimadzu Prominence system (Shimadzu, Japan) equipped with a Nucleosil C18 column (Macherey–Nagel, Germany) (150 × 4.6 mm; particle size 5 μm). As previously reported [34], the mobile phase consisted of 0.1% of formic acid in acetonitrile (A) and 0.1% of formic acid in water (B) with gradient elution (10% for B, 0 min; 90% for B, 13–15 min; 10% for B, 15–20 min). The flow rate was fixed at 0.6 mL/min, the detection wavelength was 480 nm and the retention time was 14 min. The calibration curve was established by obtaining a limit of detection (LOD = 2.8 μg/mL) and a limit of quantification (LOQ = 8.4 μg/mL), a correlation coefficient of *R*^2^ = 0.999, and an inter-day coefficient of variance < 8.5%.

Samples labeled as free DOXC_12_ and total DOXC_12_ prepared above were quantified using this HPLC method to obtain the amount of DOXC_12_ in the LNC. The encapsulation efficiency and drug loading of DOXC_12_-LNC were calculated using the following equations (*n* = 3):

1 $$\mathrm D\mathrm L\%=\frac{\mathrm{amount}\;\mathrm{of}\;{\mathrm{DOXC}}_{12}\mathrm{in}\;\mathrm{the}\;\mathrm{LNC}}{\mathrm{amount}\;\mathrm{of}\;\mathrm{oil}\;\mathrm{component}\;(\mathrm{Labrafac}^\circledR)}\times100$$

2 $$\mathrm E\mathrm E\%=\frac{\mathrm{amount}\;\mathrm{of}\;{\mathrm{DOXC}}_{12}\;\mathrm{in}\;\mathrm{the}\;\mathrm{LNC}}{\mathrm{total}\;\mathrm{amount}\;\mathrm{of}\;{\mathrm{DOXC}}_{12\;}\mathrm{initially}\;\mathrm{added}}\times100$$

#### Rheological behavior

Rheological behavior of blank LNC, blank LNC^CL^, DOXC_12_-LNC, and DOXC_12_-LNC^CL^ was evaluated using a Modular Compact Rheometer MCR 102 (Anton Paar, Austria) with a cone plate (diameter 50 mm) and setting an angle of 0.5° and a working gap at 0.051 mm. Four hundred microliters of formulations was extruded from 30-G needles and placed directly on the testing plate. The shear-strain-amplitude sweep was applied to the sample, at a constant strain amplitude of 1% to stay in the linear regime of deformation. The evolution of the storage modulus *G*′ and loss modulus *G*″ were measured as a function of the angular frequency (0.1–10 Hz) (*n* = 3) as previously reported [23].

#### Drug release

The in vitro DOXC_12_ release kinetics from the DOXC_12_-LNC^CL^ hydrogel was studied in artificial cerebrospinal fluid (aCSF) for 30 days as previously reported [23]. One hundred microliters of gel was placed at the bottom of the vial and immersed in 900 μL of aCSF at pH 7.4. The vials were incubated at 37 °C. At fixed time intervals, 100 μL of supernatant was withdrawn for further analysis and replaced by 100 μL of fresh aCSF. The samples were diluted in methanol at a ratio of 1:20 and centrifuged at 10,000 g for 15 min and DOXC_12_ was quantified by HPLC. At the last time point, the residual hydrogel was recovered and appropriately diluted in methanol and centrifugation as above for further HPLC quantification. The percentage of released drugs in DOX equivalent and DOXC_12_ equivalent was calculated based on the initial drug amount in the hydrogel (*n* = 3).

### In vitro cytotoxicity studies

#### Cell cultures

Murine glioma GL261 cell line (DSMZ, Germany) was cultured in Eagle’s Minimum Essential Medium (EMEM; ATTC, USA) supplemented with penicillin G sodium (100 U/mL) and streptomycin sulfate (100 μg/mL) (Gibco, USA) and 10% Bovine Fetal Serum (Gibco, USA). Cells were subcultured in 75 cm^2^ culture flasks (Sigma-Aldrich, USA) and incubated at 37 °C and 5% CO_2_.

#### Cytotoxicity studies in GBM cells

Cytotoxicity assays of DOXC_12_ and diluted DOXC_12_-LNC^CL^ were performed on GBM cell lines using crystal violet staining. GL261 cells were seeded at a density of 3000 cells/well in 96-well plates and incubated at 37 °C and 5% CO_2_ for 24 h. Then, cells were treated with different concentrations of DOXC_12_-LNC^CL^, DOXC_12_ (prediluted in 0.1% DMSO), and blank LNC^CL^ diluted in the culture medium and incubated for 72 h. The drug concentration range was 1–5000 nM. Blank LNC^CL^ were diluted at the same dilution range as the DOXC_12_-LNC^CL^ formulations. Cells treated with 1% Triton X-100 (Sigma-Aldrich, USA) and untreated were used as controls. At the end of the incubation period, the treatments were removed, and cells were fixed with 4% formaldehyde (Carl Roth, Germany) at room temperature for 20 min before staining with crystal violet solution for 20 min (0.5% in 20% methanol; Sigma-Aldrich, USA). The plates were rinsed three times and let dry for 2 h. Then, 100 µL of methanol was added to each well and absorbance was quantified at a wavelength of 560 nm using Omega Plate Reader (BMG Labtech, Germany). Data were normalized to be compared to the untreated group (100% viability).

#### Transfection and purification of green fluorescent protein–expressing GL261 cells (GL261-GFP)

GL261 cells expressing green fluorescent protein (GFP) were obtained through transfecting GL261 cells with DNA plasmid pEGFP-C1 (Clontech, USA). Regular cultured GL261 cells were harvested in a native culture medium when they reached 80% confluence. Cells were incubated with 8 ng/mL of pEGFP-C1 plasmid and 20 µg/mL of Lipofectamine 2000 (Invitrogen, USA) to increase the transfection efficiency by lipofection. After 4 h, the culture medium was aspirated and replaced with a complete culture medium supplemented with 0.8 mg/mL of Geneticin™ Selective Antibiotic (Gibco, USA). After the establishment of a stable cell pool resistant to the antibiotic, GL261-GFP cells were sorted by flow cytometry (Beckman Coulter, USA) and subcultured in 75-cm^2^ culture flasks (Sigma-Aldrich, USA) and incubated at 37 °C and 5% CO_2_.

#### Preparation of fluorescent GBM spheroids

GL261-GFP cells were harvested when they reached 80% confluence. Cells were resuspended in a complete medium supplemented with 20% methylcellulose (Thermo Fisher, USA) at a concentration of 15,000 cells/mL. Three thousand cells were seeded in a round-bottom 96-well plate and incubated for 24 h at 37 °C and 5% CO_2_. The formation of round green fluorescent spheroids was confirmed by microscopy (Leica, Germany).

### Ex vivo anti-cancer study by organotypic brain slice culture model

#### Preparation of organotypic brain slices

Six to 8-week-old C57BL6J female mice (Envigo, France) were anesthetized by intraperitoneal injection of ketamine/xylazine (100 mg/kg and 13 mg/kg, respectively). Mice were perfused with 10 mL Dulbecco’s phosphate-buffered saline (DPBS) for 10 min. Then, the brain was collected and stored in a DPBS solution supplemented with an antibiotic–antimycotic solution (Anti-Anti; 200 U/mL of penicillin, 200 µg/mL of streptomycin, and 25 µg/mL of Gibco amphotericin B; Gibco, France) on ice. After the removal of the cerebellum, the flat side of the brain was glued to the flat surface of agarose gel (4%). The gel is further fixed to an operating flat plate of a vibratome (Leica VT1200 S) with a tank filled with DPBS solution supplemented with Anti-Anti as before. The brain was sliced with a thickness of 250 μm by a blade at a fixed speed and frequency. Intact brain slices were collected in Petri dishes filled with DPBS solution supplemented with Anti-Anti as before on ice. Under the laminar flow hood, slices were transferred on cell culture inserts (pore size 0.4 µm, Merck Millipore, USA) wetted in brain-culture medium (3 slices per insert). The medium was composed of 50% of DMEM (4.5 g/l glucose + glutamine + pyruvate; Gibco, France) supplemented with penicillin G sodium (200 U/mL) and streptomycin sulfate (200 μg/ mL) (Gibco, USA), 25% of horse serum, and 25% of Hank’s Balanced Salt Solution (Gibco, France). Using forceps, the inserts were gently placed in 6-well plates (Greiner, France) and 1.2 mL of brain-culture medium was inserted at the bottom of the well. The brain slices were incubated at 37 °C and 5% CO_2_. The medium was changed every 2 days.

### Ex vivo anti-cancer efficacy of DOXC_12_-LNC^CL^ in an organotypic brain slice culture model

The GL261-GFP spheroids that reached a diameter of 0.3 mm were carefully transferred on top of the organotypic brain slices using a 200-µL tipped pipette (one spheroid per organotypic brain slice) and incubated at 37 °C and 5% CO_2_. Twenty-four hours after spheroid inoculation, brain organotypic slice bearing GL261-GFP spheroids were treated with 1 μM of DOXC_12_ (prediluted in 0.1% of DMSO) and 1 μM of DOXC_12_-LNC^CL^ solution or blank LNC^CL^, appropriately diluted in the brain-culture medium. The organotypic slices were cultivated at 37 °C and 5% CO_2_ for 2 weeks and the drug-containing brain-culture medium was replaced every 2 days. The slices were scanned using a fluorescence LSM800 inverted microscope (Zeiss, Germany) with excitation filters of GFP to evaluate the presence and monitor the size of the spheroids. Data were normalized to time zero and fluorescence intensity was quantified by ImageJ software (Fiji software, v.1.53f51).

### In vivo studies: anti-cancer efficacy of DOXC_12_ in orthotopic GL261-bearing models

#### GL261 tumor orthotopic grafting in mouse

For the intracranial GBM cell grafting, 6-week-old C57BL6J female mice (Charles River, France) were anesthetized by intraperitoneal injection of ketamine/xylazine (100 mg/kg and 13 mg/kg, respectively). To hydrate the animal, 200 µL of 0.9% sodium chloride (Aguettant, France) was injected subcutaneously into the flank. Mice were then fixed on a stereotactic frame, the head was disinfected with an antiseptic solution (Vétédine^®^ solution, Vetoquinol, Lure, France), 20–30 µL of lidocaine (10 mg/mL, Aguettant, France) was injected subcutaneously on the head, and the eyes were protected with an ophthalmic gel (Ocry-gel, TVM lab, Lempdes, France). Then, a 3–5-mm-long incision was made along the midline. A burr hole was drilled into the skull at the right frontal lobe, 0.5 mm posterior and 2.1 mm lateral to the bregma, using a high-speed drill (Tack Life Tools, USA; 0.8 mm diameter round end engraving burrs: Dremel, the Netherlands). A 10-µL 26S-gauge syringe with a cemented 51-mm needle (Hamilton, Rungis, France) was used to inject 1 × 10^5^ GL261 cells suspended in 2 µL of culture medium at a depth of 2.1–2.5 mm from the outer border of the brain, using an osmotic pump PHD 2000 infusion (Harvard Apparatus, France) at a speed of 0.7 µL/min. The wound was then closed using tissue adhesive glue (3M Vetbond^®^, Cergy-Pontoise, France), and the animals recovered under an infrared heating lamp. The presence and location of the tumors were confirmed randomly in at least half of the animals on the day before treatment by magnetic resonance imaging using a protocol previously published in our lab [38] (data not shown).

#### In vivo anti-cancer efficacy of DOXC_12_

To evaluate the anti-cancer efficacy of DOXC_12_, we tested two different intratumoral convection-enhanced delivery (CED) treatment regimens (varying time of treatment and dose). After tumor inoculation, mice were randomly assigned to three groups: (1) untreated: the animals did not receive any treatment (*n* = 17); 2) low-dose/late treatment: the animals were treated at a dose of 2.5 mg/kg DOXC_12_ (administration of 5 μL intratumorally by CED; 10 mg/mL in 5% of DMSO) at day 15 post-tumor grafting (*n* = 8); and (3) high-dose/early treatment: the animals were treated at a dose of 3.75 mg/kg DOXC_12_ (administration of 7.5 μL intratumorally by CED; 10 mg/mL in 5% of DMSO) at day 11 post-tumor grafting (*n* = 9). On the day of the treatment, all mice were anesthetized with ketamine/xylazine (100 mg/kg and 13 mg/kg, respectively). As previously described, mice were hydrated with a physiological solution and disinfected with an antiseptic solution and a local anesthetic was injected at the site of the incision. The mice were then fixed on a stereotactic frame and the skin was opened again. For animals of groups 2 and 3, a 10-µL Hamilton syringe preloaded with the right amount of treatment was inserted in the previous cranial hole at the same depth. An osmotic pump PHD 2000 infusion (Harvard Apparatus, France) was used to inject the treatment at a constant speed of 0.7 µL/min. After treatment, the wound was closed using a tissue adhesive glue and the animals recovered under an infrared heating lamp. Mice were then monitored daily, and their body weight was measured every 2–3 times per week. The mice were sacrificed by dislocation when an endpoint (20% body weight loss or 10% body weight loss plus clinical signs of distress, e.g., paralysis, arched back, or lack of movement) was reached.

### Anti-tumor efficacy of DOXC_12_-LNC^CL^ hydrogel in an orthotopic GL261 GBM-resected model

To evaluate the anti-cancer efficacy of DOXC_12_**-**LNC^CL^ hydrogel in clinically relevant conditions, we used a preclinical model previously developed and validated in our lab to resect GBM tumors [3, 39]. In this model, animals were grafted with GL261 cells as described above. At day 14 post-tumor inoculation, all mice received tumor resection and were randomly assigned to one of 5 following groups: (1) untreated (*n* = 8); (2) blank LNC^CL^ hydrogel (*n* = 6); (3) ibuprofen (*n* = 8); (4) DOXC_12_-LNC^CL^ hydrogel (*n* = 9); and (5) ibuprofen and DOXC_12_-LNC^CL^ hydrogel (*n* = 8). Ibuprofen was administered by intraperitoneal injection at a dose of 30 mg/kg (120 μL of ibuprofen Pedea^®^ solution, 5 mg/mL, France). The ibuprofen treatment was performed post-surgery and every 24 h for 3 days (4 administrations in total). The DOXC_12_-LNC^CL^ and blank LNC^CL^ treatments were injected into the tumor resection cavity at the time of surgery. The DOXC_12_ dose was 5 mg/kg (5 μL of DOXC_12_-LNC^CL^ hydrogel, 20 mg/mL).

For the tumor surgery, all mice were anesthetized with ketamine/xylazine (100 mg/kg and 13 mg/kg, respectively) and then fixed on a stereotactic frame. The skin was opened on a previous surgical scar and the periosteum was removed revealing the previous burr hole. A high-speed drill was used to gently break the skull around the burr hole, after which fine-tip tweezers (Dumont, Switzerland) were used to obtain a circular cranial window exposing the brain. A biopsy punch (2 mm Ø, Kai Medical, Germany) was inserted 2 mm deep and twisted to cut the brain/tumor tissue. Once withdrawn, a Pasteur pipette connected to a vacuum pump (Vacuubrand GMBH + CO KG, Germany) sucked the explant. Residual blood in the surgery cavity was removed using an absorbable hemostatic triangle (Fine Science Tools, Germany). For groups 1 and 3, no treatment was administered in the tumor cavity. For groups 2, 4, and 5, blank LNC^CL^ hydrogel or DOXC_12_-LNC^CL^ hydrogel was injected into the resection cavity using a 0.3-mL insulin syringe. Then, the dural window was repaired by covering it with a 4 × 4 mm square piece of Neuro-Patch^®^ (Aesculap, Germany) impregnated with fibrin sealant (TISSEEL PRIMA; Baxter, France). The wound was then closed using tissue adhesive glue. Mice were then monitored daily, and their body weight was measured every 2–3 times per week. The mice were sacrificed by dislocation when they reached the endpoints.

### Statistics

Statistical analysis was performed using GraphPad Prism (GraphPad Software, USA) for ANOVA assay for cellular and ex vivo studies. *p*-values < 0.05 were considered statistically significant (*p < 0.05, **p < 0.01, ***p < 0.001, and ****p < 0.0001). IC_50_ values were calculated by performing a non-linear regression inhibition *vs* response variable slope analysis (*n* = 3) in GraphPad Prism. In the experiments, *n* corresponds to the number of independent experiments performed. For the in vivo efficacy studies, the statistical analysis was estimated from a comparison of Kaplan–Meier survival curves using the log-rank test (Mantel-Cox test).

## Results and discussion

### Anti-cancer efficacy of DOXC_12_ in an orthotopic GBM model

**Fig. 2:**
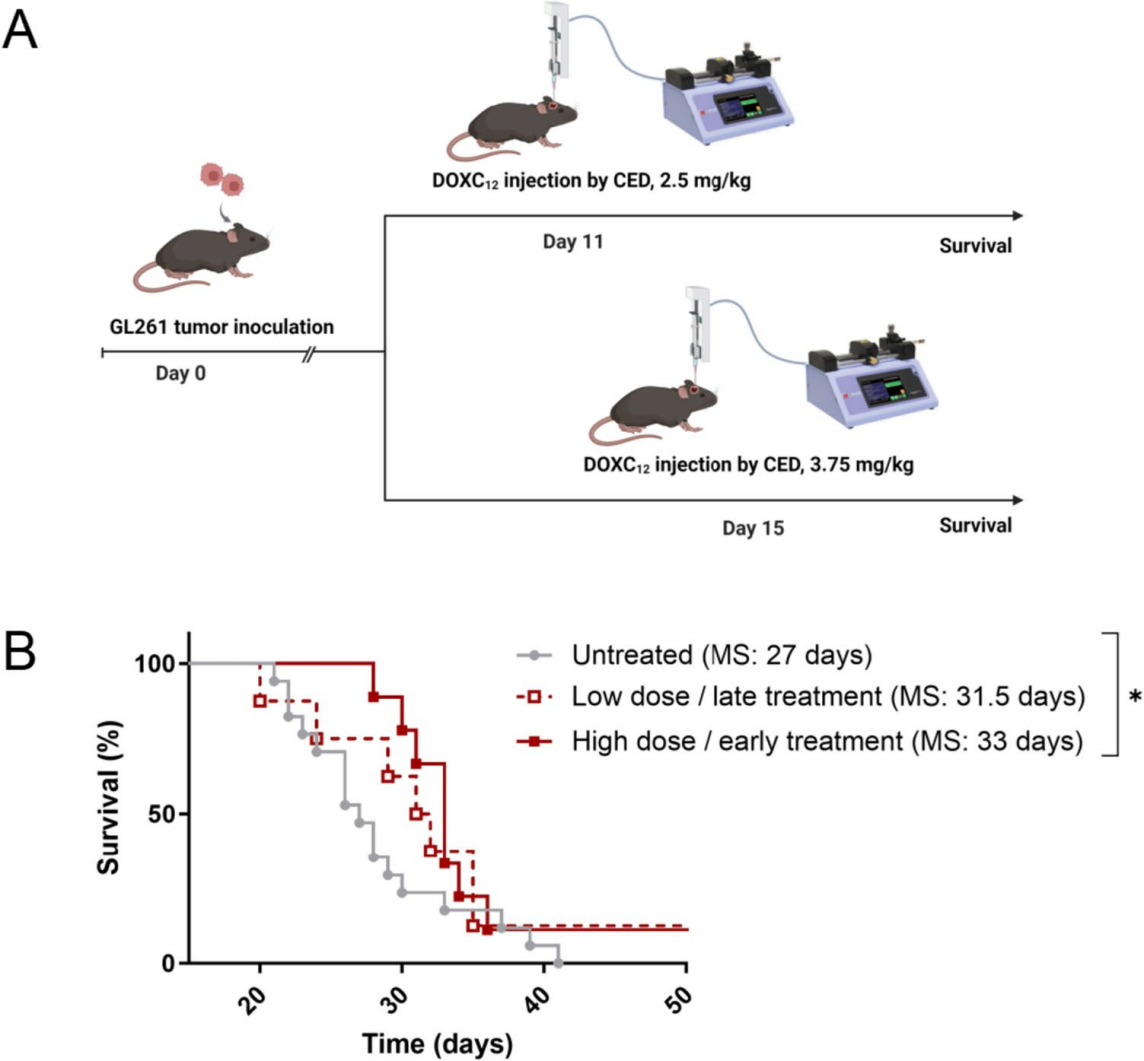
Proof-of-concept studies to evaluate the anti-tumor efficacy of prodrug DOXC_12_ in orthotopic GL261-bearing mice. **A** Schematic representation of the therapeutic regimens tested in an orthotopic GL261 mouse model. DOXC_12_ was intratumorally administered by CED at a dose of 2.5 mg/kg (50 μg/mouse) on day 15 or at a dose of 3.75 mg/kg (75 μg/mouse) on day 11. **B** Kaplan–Meier survival curves of mice after intratumoral administration of low dose/late treatments or high-dose/early treatment regimen with DOXC_12_ (*n* = 8–17) (MS median survival; *p < 0.05)

To develop an injectable LNC-based hydrogel loaded with the anti-cancer drug DOX, the amphiphilic prodrug DOXC_12_ with a hydrazone linker was synthesized. In previous work, we showed that DOXC_12_ was quickly internalized in GBM cells and had an enhanced cytotoxic effect in vitro on GL261 cells and U87-MG cells compared to DOX [34]. Here, we evaluated its anti-cancer efficacy in vivo in an orthotopic GL261 GBM mouse model at two different drug doses at two different time points post-tumor inoculation (Fig. 2A). Administering a dose of DOXC_12_ of 50 μg/mouse (37 μg/mouse of DOX in equiv.) at day 15 post-inoculation led to a slight delay in tumor growth but no significant difference in survival was observed compared to untreated animals (31.5 *vs* 27 days, respectively). Administering a dose of 75 μg/mouse DOXC_12_ (55 μg/mouse of DOX in equiv.) at an earlier time (day 11 post-inoculation) increased the therapeutic efficacy of the treatment with a significant increase in median survival compared to untreated animals (33 *vs* 27 days, respectively; *p < 0.05, Fig. 2B). In both treatment regimens, the treatment led to a long-term survivor. These results indicate that DOXC_12_ has a dose-dependent anti-cancer efficacy which is consistent with previously reported cytotoxicity in GBM cells [34]. These injections of DOXC_12_ did not induce detectable side effects as no loss of body weight nor change in animals’ behavior was observed.

The doses employed in this study were selected to achieve a therapeutic effect of DOXC_12_ while mitigating the risk of severe side effects. Previous investigations of local DOX delivery in various GBM models have yielded mixed results. For instance, in an orthotopic U87-Luc GBM model, a local administration of 10 µL of DOX (equivalent to 4 μg/mouse or 20 µg/kg) via CED injection on day 11 post-tumor inoculation did not significantly prolong survival [40]. Similarly, in an orthotopic GL261 model, local DOX administration at a dose of 5 µg/mouse via CED also failed to significantly extend the median survival time compared to the untreated group [41]. Striking a balance between effective therapeutic outcomes and the maximum tolerated dose, Graham-Gurysh et al. conducted dose evaluations of DOX in healthy brains, revealing that 200 μg/mouse induced significant weight loss in animals, while 100 µg/mouse was well-tolerated [31]. However, it is important to note that in our study, DOXC_12_ does not provide a sustained drug release without a scaffold, unlike the acetylated dextran scaffold used in their study. Given this difference, we proceeded to evaluate the anti-cancer efficacy of DOXC_12_ in our study using two safe doses (50 and 75 μg/mouse) in an orthotopic GL261 model.

Once demonstrated that DOXC_12_ could induce a delay in tumor growth after local administration in the brain, we proceeded to the development of a drug delivery system able to fit in the post-surgical cavity and provide a sustained drug release over time. The release profile and rheological properties of the LNC-based hydrogels depend on the amount of drug loaded into the system [24, 42]. To determine the appropriate amount of DOXC_12_ to be loaded into a DOXC_12_-LNC hydrogel, we considered several key factors, including the previously reported toxicity-free dose of DOX (100 μg/mouse), the indicated effective dose of DOXC_12_ (75 μg/mouse, equivalent to 55 μg/mouse of DOX), and the expected sustained drug release from the hydrogel. Additionally, considering the maximum amount that can be injected into post-surgical cavities in mice (5 µL) [25, 26], we formulated DOXC_12_-LNC with a drug loading of 9% (100 μg/mouse of DOXC_12_). By considering these factors, we aimed to achieve an appropriate drug concentration for targeted treatment, optimizing therapeutic outcomes while minimizing the potential for adverse effects.

### Optimization of the rheological behavior of the DOXC_12_-LNC hydrogel

An injectable system for the post-surgical GBM cavity should ideally present mechanical properties compatible with the brain tissue (~ 1.7 kPa) to avoid excessive intracranial pressure and because stiffness can impact GBM cell progression, proliferation, and invasion [43, 44]. DOXC_12_-LNC hydrogel was prepared using the phase-inversion technique method. DOXC_12_ was synthesized as an amphiphilic prodrug aiming to increase drug loading in the LNC and form a hydrogel by H-bonds between LNC molecules. Moreover, the presence of a pH-sensitive linker could support the release of the drugs and the degradation of the gel over time.

**Fig. 3:**
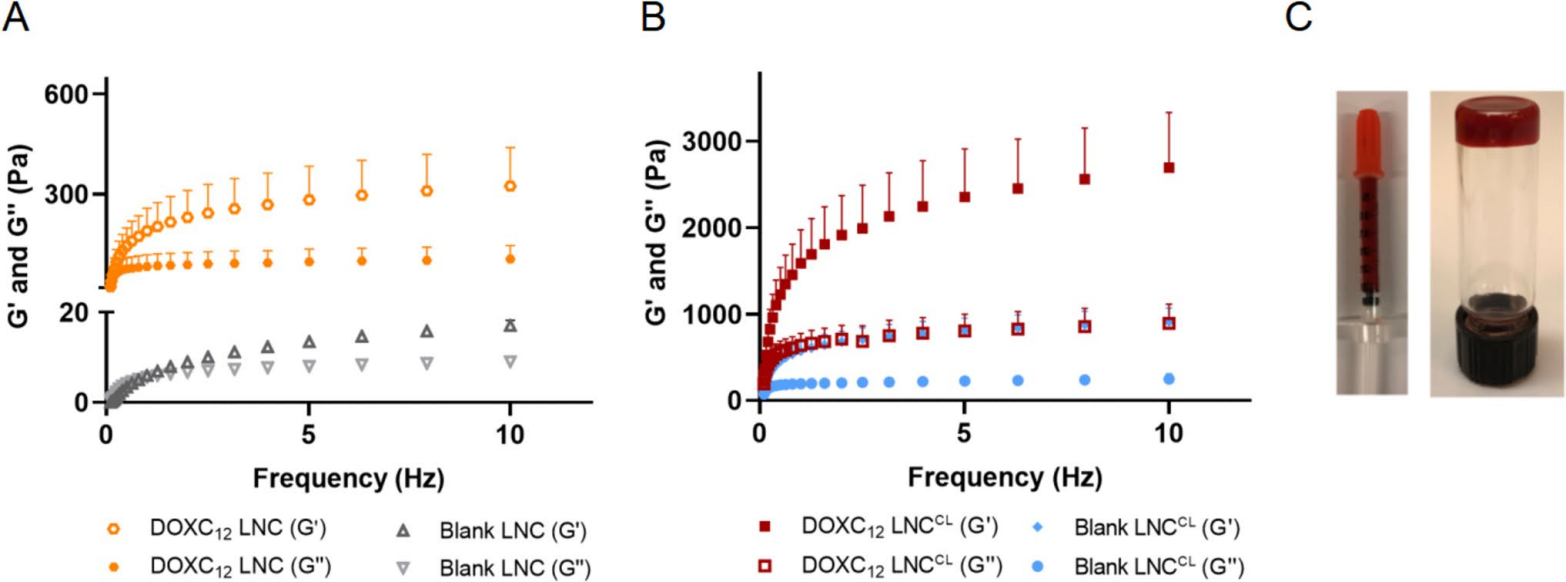
Rheological behaviors of the formulations. **A** Storage modulus (*G*′) and loss modulus (*G*″) of blank LNC and DOXC_12_-LNC hydrogel. **B** Storage modulus (*G*′) and loss modulus (*G*″) of blank LNC^CL^ and DOXC_12_-LNC^CL^ hydrogel cross-linked by CytC_16_ (3.4%, w/w). Storage and loss modulus *vs* frequency (Hz) were measured (mean ± SD, *n* = 3). **C** Injectable DOXC_12_-LNC^CL^ hydrogel was stored in an insulin syringe and a reverted vial

**Fig. 4:**
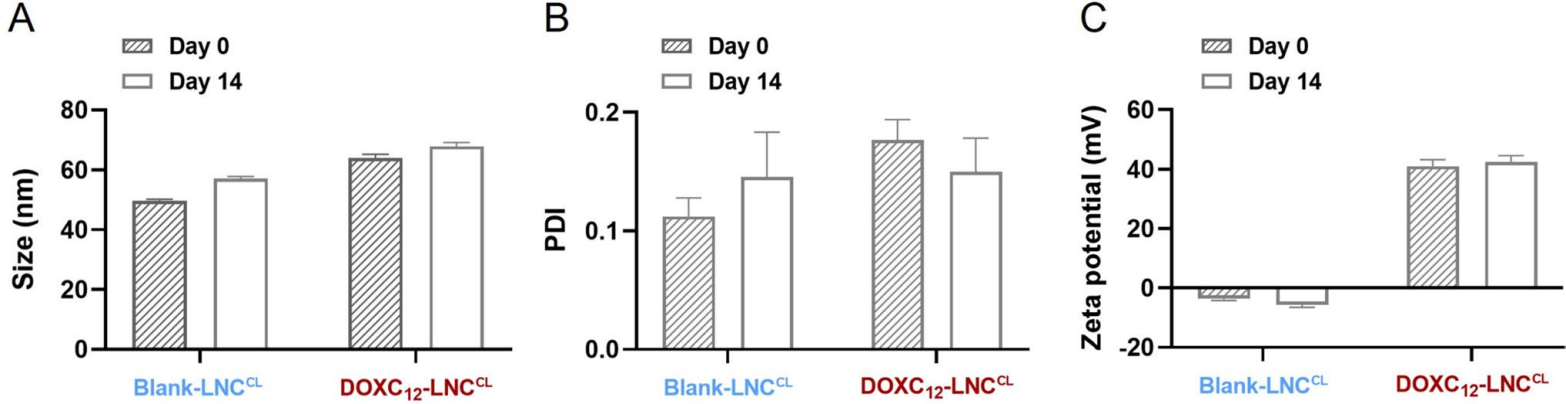
Short-term physical stability of DOXC_12_-LNC^CL^ hydrogel at 4 °C after 2 weeks: LNC size (**A**), PDI (**B**), and zeta potential (**B**). Data was compared with day 0 (mean ± SD, *n* = 3)

As previously observed with GemC_12_-LNC and CytC_16_-LNC hydrogels, the drug loading of DOXC_12_ could influence gelation time, rheology, and degradation kinetics due to its function as a cross-linking agent [23, 24, 42]. The rheological behavior of blank LNC and DOXC_12_-LNC hydrogel (9%, w/w) without using any cross-linking agent was first evaluated by measuring storage modulus *G*′ (elastic behavior) and loss modulus *G*″ (viscous behavior). As illustrated in Fig. 3A, blank LNC formulation was a solution without an elastic behavior while DOXC_12_-LNC showed gel properties with storage modulus consistently higher than loss modulus. Initially, the gel formed by DOXC_12_-LNC with a drug loading of 9% and a DOXC_12_ concentration of 20 mg/mL was found to be too soft to remain intact in the resection cavity (Fig. 4B). The final storage modulus *G*′ of DOXC_12_-LNC hydrogel reached 0.28 ± 0.03 kPa indicating the spontaneous gelation ability of DOXC_12_-LNC hydrogel due to the H-bonds of DOX moieties in the surface of LNC. However, the DOXC_12_-LNC hydrogel with a fixed drug loading (9%, w/w) cannot reach a brain storage modulus [13], and cerebral implants softer than brain tissue can lead to poor material stability and fixation at the implant site followed by reduced therapeutic efficacy [45, 46].

To address this issue and retain the targeted drug concentration, CytC_16_ was added during the LNC formulation process as a cross-linking agent, resulting in the DOXC_12_-LNC^CL^ formulation with a cerebral injection-adapted mechanical behavior. CytC_16_ at 3.4% w/w was incorporated into the formulation to enhance the stiffness of the hydrogel (DOXC_12_-LNC^CL^). Both blank LNC^CL^ and DOXC_12_-LNC^CL^ hydrogel showed a typical rheological behavior of solid-like materials where the storage modulus dominates over loss modulus *G*″ as a function of frequency (Fig. 3B). Compared to blank LNC^CL^ (final storage modulus *G*′ *vs* loss modulus *G*″, 0.91 ± 0.16 kPa *vs* 0.25 kPa ± 0.06 kPa), the final storage modulus *G*′ (2.70 ± 0.64 kPa) and loss modulus *G*″ (0.89 ± 0.22 kPa) of DOXC_12_-LNC^CL^ are increased. Indeed, DOX moieties and cytidine moieties oriented toward the water phase on the surface of LNC can form H-bonds cross-linking and immobilizing the water phase to form a gel. In the following studies, only blank LNC^CL^ and DOXC_12_-LNC^CL^ hydrogel cross-linked with CytC_16_ (3.4%, w/w) were investigated.

### Physicochemical characterization of DOXC_12_-LNC hydrogels

Both DOXC_12_-LNC and DOXC_12_-LNC^CL^ formulations were characterized by a fast sol–gel transition at room temperature after shock dilution with water at the end of the last cooling cycle of the formulation process (within 5–7 min). As shown in Table 1, when properly resuspended in water, both formulations displayed a size around 60 nm with a narrow size distribution (PDI < 0.2) and a high drug encapsulation efficiency of approximately 95%.

**Table 1:** Size distributions (*Z*-average size, *PDI* polydispersity index) and surface charge (*ζ* potential) for LNCs (*n* = 3, mean ± SD)

	Size (nm)	PDI	ζ potential (mV)	EE%	DL%
Blank LNC	50 ± 2	0.05 ± 0.01	−3 ± 1	/	/
Blank LNC^CL^	60 ± 5	0.06 ± 0.02	−4 ± 1	/	/
DOXC_12_-LNC	57 ± 3	0.15 ± 0.02	22 ± 2	95 ± 4	9 ± 1
DOXC_12_-LNC^CL^	65 ± 1	0.07 ± 0.02	12 ± 1	95 ± 2	9 ± 2

The drug loading of DOXC_12_-LNC was 9% w/w, corresponding to a concentration of 20 mg/mL of DOXC_12_ in the formulation (14.7 mg/mL of DOX equiv.). These improvements in size and drug loading surpassed the previously reported DOX freebase LNC in liquid form, which had a size of over 200 nm and a low drug content of approximately 0.1 mg/mL [47]. Notably, while blank LNC exhibited a negative surface charge (− 3 mV), DOXC_12_-LNC showed a positive surface charge (+ 22 mV) due to the presence of DOX moieties containing an amine group on the LNC surface. As previously observed with GemC_12_-LNC and CytC_16_-LNC, the hydrogel formation is likely facilitated by interactions between the hydrophilic DOX moieties on the exterior of the LNC, while the lipophilic -C_12_ alkyl chains are oriented toward the oil core, thereby anchoring the molecule to the LNC structure. DOXC_12_-LNC^CL^ showed a slightly less positive surface charge compared to DOXC_12_-LNC possibly due to the interaction between the cytidine moiety of CytC_16_ and the DOX moiety of DOXC_12_ on the LNC surface. This positive surface charge contributes to a repulsive force between LNCs, enhancing the physical stability of the colloidal system.

The stability of DOXC_12_-LNC^CL^ and blank LNC^CL^ formulations was evaluated for 14 days post-storage in syringes at 4 °C. No significant differences were observed in terms of size (Fig. 4A), PDI (Fig. 4B), and zeta potential (Fig. 4C) of formulations, which is consistent with previously reported high stability of unloaded LNC and drug-loaded LNC [42, 48].

### DOXC_12_ release from DOXC_12_-LNC^CL^ hydrogel

**Fig. 5:**
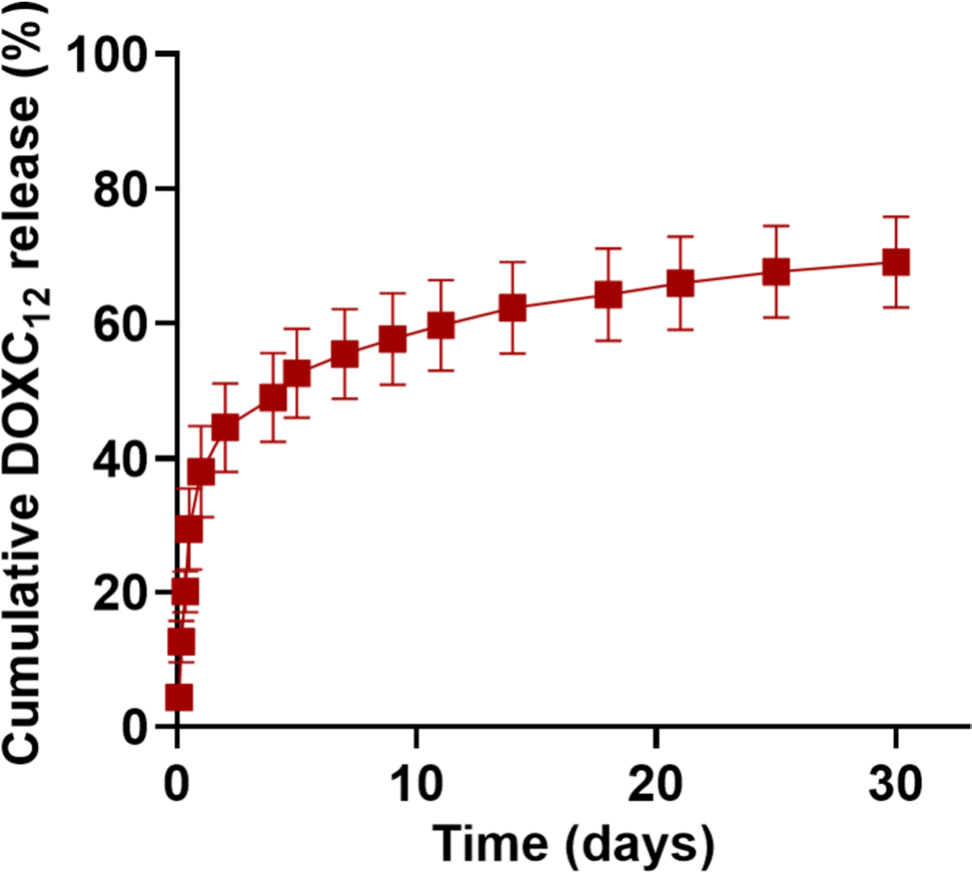
In vitro cumulative release behavior of DOXC_12_-LNC^CL^ hydrogel. The release study was performed in aCSF medium (pH 7.4) at 37 °C and the cumulative drug was quantified by HPLC (mean ± SD, *n* = 3)

The in vitro release kinetics of DOXC_12_ and DOXC_12_-LNC from the DOXC_12_-LNC^CL^ hydrogel were assessed in aCSF medium (pH 7.4) at 37 °C and the cumulative drug release was quantified using HPLC. For this experiment, a dose of hydrogel corresponding to 1.52 ± 0.06 mg of DOX equiv. was used. The results revealed that an initial burst release of DOXC_12_ from DOXC_12_-LNC^CL^ hydrogel reached 45 ± 7% (0.67 ± 0.10 mg of DOX equiv.) within the first 48 h, followed by a release up to 62 ± 7% (0.84 ± 0.10 mg of DOX equiv.) at 2 weeks and 69 ± 7% (1.05 ± 0.10 mg of DOX equiv.) at 1 month (Fig. 5). By the end of the experiment, the hydrogel retained 23 ± 3% (0.34 ± 0.04 mg of DOX) of the drug with a total drug recovery of approximately 92 ± 7% (1.39 ± 0.11 mg of DOX).

Consistent with previous observations, the drug release behavior of the non-polymeric hydrogel (DOXC_12_-LNC^CL^) is likely influenced by two distinct mechanisms. Initially, DOXC_12_ located on the surface of the hydrogel may exhibit fast diffusion into the drug release medium, resulting in an initial burst release of the drug [49]. This rapid-release phase allows for the immediate exposure of the drug to the surrounding environment, potentially targeting and affecting nearby residual tumor cells at the site of the post-surgical cavity. Following this initial release of 48 h, the DOXC_12_-LNC^CL^ hydrogel demonstrated nearly zero-order drug release behavior (*R*^2^ = 0.91), indicating sustained drug release over the subsequent weeks. This can be attributed to the gradual degradation of the LNCs that form the hydrogel. As the LNCs degrade, they continue to release DOXC_12_ in a controlled and sustained manner [50]. This sustained drug release mechanism allows for prolonged and controlled drug exposure, potentially targeting and eliminating far-infiltrating tumor cells over an extended period. By combining both the initial burst release and the subsequent sustained release, it is promising to be applied as a local drug delivery system for the treatment of GBM during the period between surgery and subsequent chemoradiation (typically 4–6 weeks).

### DOXC_12_-LNC^CL^ show superior cytotoxicity than DOXC_12_ in GL261 cells

**Fig. 6:**
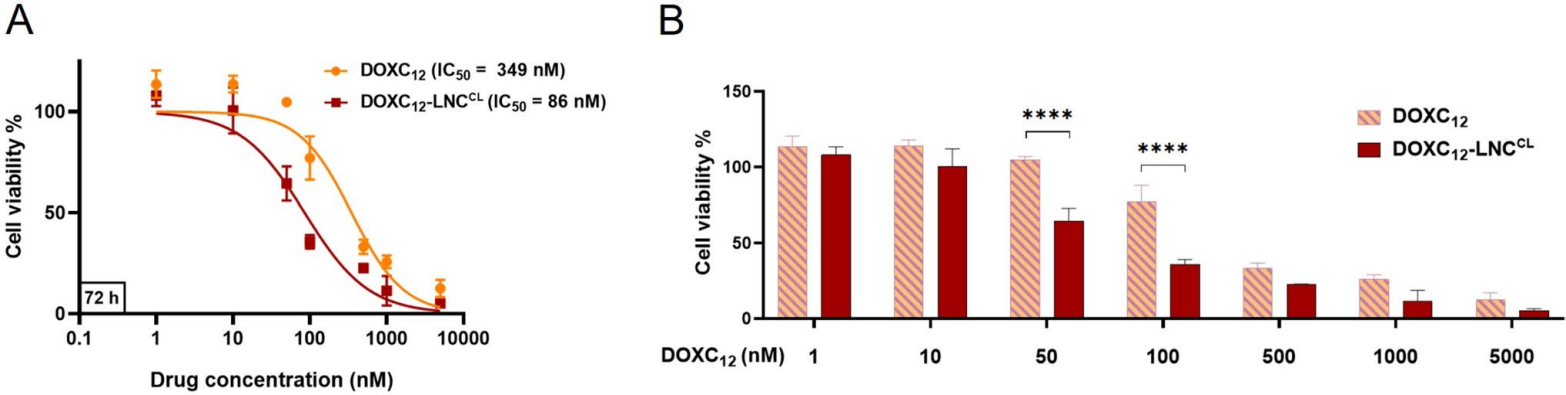
Cytotoxicity of DOXC_12_ and DOXC_12_-LNC^CL^ in GL261 cells for 72 h. The cytotoxic effect of the treatments was assessed by crystal violet assay and presented in curve graph (**A**) and bar graph (**B**). The percentage of cell survival is compared to untreated cells (assumed as 100%) (mean ± SD, *n* = 3). Statistical analyses were performed using unpaired *t*-test for the IC_50_ values (**A**) and two-way ANOVA with Bonferroni’s multiple comparisons test for bar graph (**B**) (**p < 0.01, ****p < 0.0001)

In vitro cytotoxicity of DOXC_12_-LNC^CL^ was evaluated on GL261 GBM cells by crystal violet staining after 72 h of incubation. The results are illustrated in Fig. 6 at different concentrations of DOXC_12_ (1–5000 nM). Both DOXC_12_ and DOXC_12_-LNC^CL^ induced concentration-dependent cytotoxicity on GL261 cells, whereas the IC_50_ value of DOXC_12_-LNC^CL^ (IC_50_ = 86 nM) was significantly decreased compared to that of DOXC_12_ (IC_50_ = 349 nM) (**p < 0.01). This result is consistent with previously reported cytotoxicity of DOXC_12_ in GL261 cells [34]. Notably, at concentrations 50 and 100 nM, DOXC_12_-LNC^CL^ exhibited significantly higher cytotoxicity compared to free DOXC_12_ (****p < 0.0001). The positive surface charge of DOXC_12_-LNC^CL^ might contribute to increased cellular uptake of DOXC_12_, as the positively charged LNCs may be attracted to the negatively charged cell membrane, particularly cancer cells with exposed negatively charged phosphatidylserine [51, 52]. Additionally, the enhanced cytotoxicity of the LNC formulations could be attributed to the presence of its key component HS15, which has been shown to have a P-gp suppressing effect, thereby inhibiting the efflux of the P-gp substrate DOX [53]. However, further investigations are required to fully elucidate the precise mechanisms of DOXC_12_-LNC^CL^ uptake by the cells. Consistent with previously reported studies on blank LNC [23, 50], no significant reduction in cell viability was observed after 72 h of culture with the unloaded LNC^CL^ at all tested concentrations, except at the highest concentration (5 μM) which led to a loss of cell viability (~ 30%). This indicates that the blank LNC^CL^ has minimal cytotoxicity on the GBM cells.

### Anti-cancer efficacy of DOXC_12_-LNC^CL^ on 3D GBM spheroids on brain slices

**Fig. 7:**
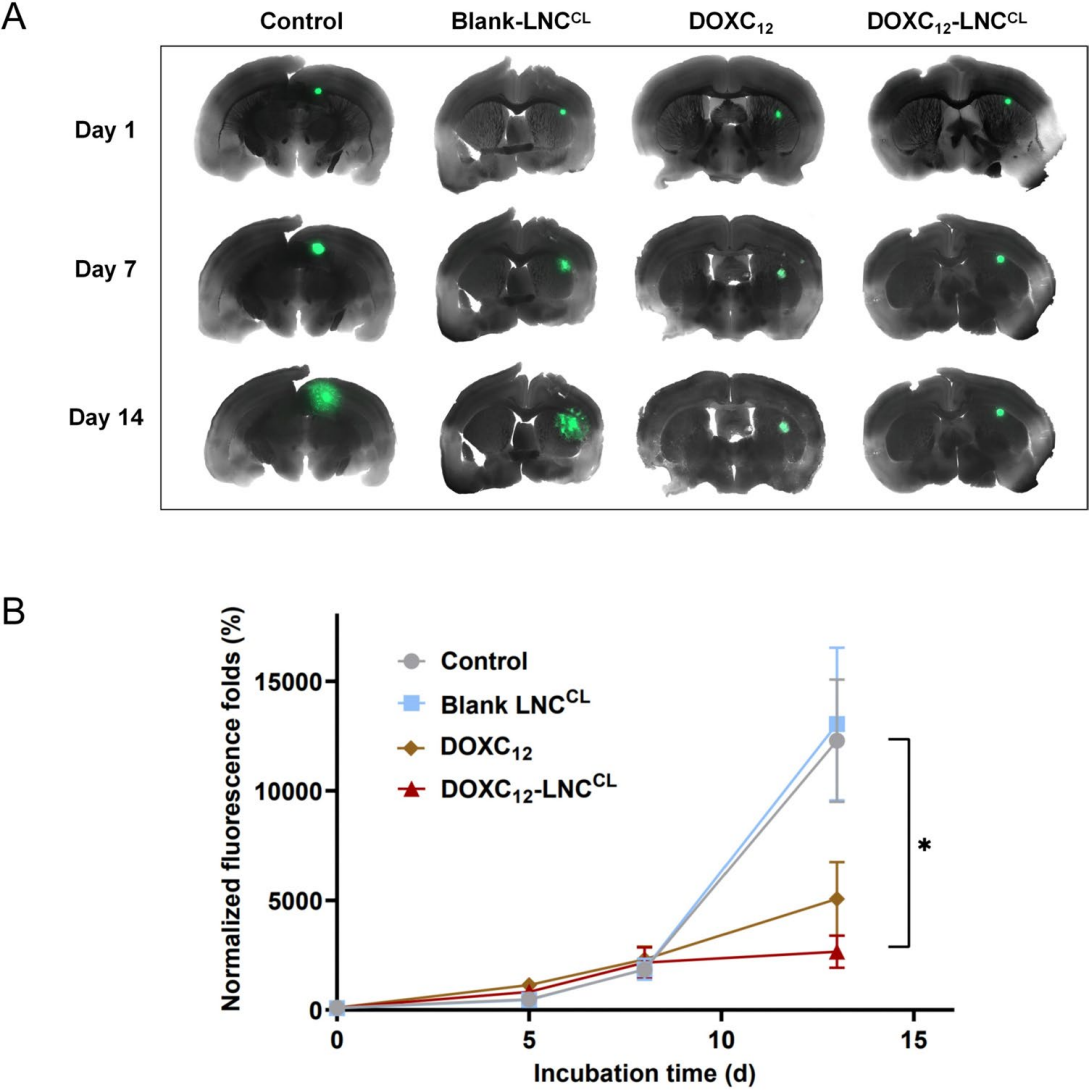
Ex vivo anticancer efficacy of DOXC_12_-LNC^CL^ in mouse organotypic brain slice model bearing GL261-GFP spheroids. **A** Images of mouse organotypic brain slices bearing GL261-GFP spheroids captured through fluorescent microscopy over time. **B** Fluorescence intensity of GL261-GFP the spheroids measured by fluorescent microscopy at different time points. Data were normalized to the initial intensity (time zero) and were reported as mean ± SEM using ImageJ software (*n* = 4). Statistical analyses were performed using one-way ANOVA with Tukey’s multiple comparisons test (*p < 0.05)

The use of a 3D tumor growth models on organotypic tissues provides a valuable model that closely mimics the morphological and functional features of the in vivo tissues, bridging the gap between 2D cell cultures and animal tumor models [54, 55]. Organotypic slice cultures represent a tractable and robust model, less expensive and less time-consuming than in vivo models, with a great potential to unravel GBM pathophysiology and drug discovery [56–58]. Moreover, they allow the reduction of the number of animals to be used to evaluate the safety and efficacy of drugs and delivery systems. While preserving the brain cytoarchitecture, the GBM organotypic co-culture system is an ex vivo model able to bring cellular heterogeneity and mimic the tumor microenvironment. In addition, the tumor-bearing organotypic brain slice model represents a versatile and robust technique that allows investigation of tumor growth of grafted spheroids that interact with resident glial cells (e.g., astrocytes and microglia) composing the brain microenvironment [59]. Indeed, slice cultures conserve the presence of vessels [60], microglial cells [61, 62], and astrocytes [63]. Organotypic slice cultures overcome some of the difficulties of in vivo studies as they provide ex vivo access to brain tissue architecture, while still enabling direct observation of the tumor growth reduction [64]. This model is also highly used to study the effect of drugs, genes or proteins present in tumor cells or in the microenvironment, or for GBM cell migration and invasion studies [65, 66]. Unlike 2D models, the viability of organotypic brain slices can be maintained for weeks, making them well-suited for evaluating the long-term anti-cancer efficacy of the tested system [67, 68]. Here, we used an ex vivo model using mouse organotypic brain slices bearing GL261-GFP spheroids to assess the anti-cancer efficacy of DOXC_12_-LNC^CL^ for 2 weeks. In the first week, the spheroids displayed a slow growth trend, with no significant differences observed among the different treatments (Fig. 7A, B). However, after 2 weeks of treatment, DOXC_12_-LNC^CL^ demonstrated a remarkable inhibition of tumor spheroid growth compared to the untreated controls or blank LNC^CL^ (*p < 0.05). Spheroids treated with DOXC_12_ alone showed a reduction in size, but the difference was not statistically significant compared to the untreated and blank LNC^CL^ controls. Importantly, no fluorescence intensity difference was observed between the spheroids treated with blank LNC^CL^ and the untreated controls, indicating that blank LNC^CL^ did not exert any significant impact on spheroid growth. These results further support the potent anticancer efficacy of DOXC_12_-LNC^CL^ in our ex vivo model and highlight its potential for long-term treatment against tumor recurrence.

### Antitumor efficacy of DOXC_12_-LNC^CL^ hydrogel on GBM-resected animal model

**Fig. 8:**
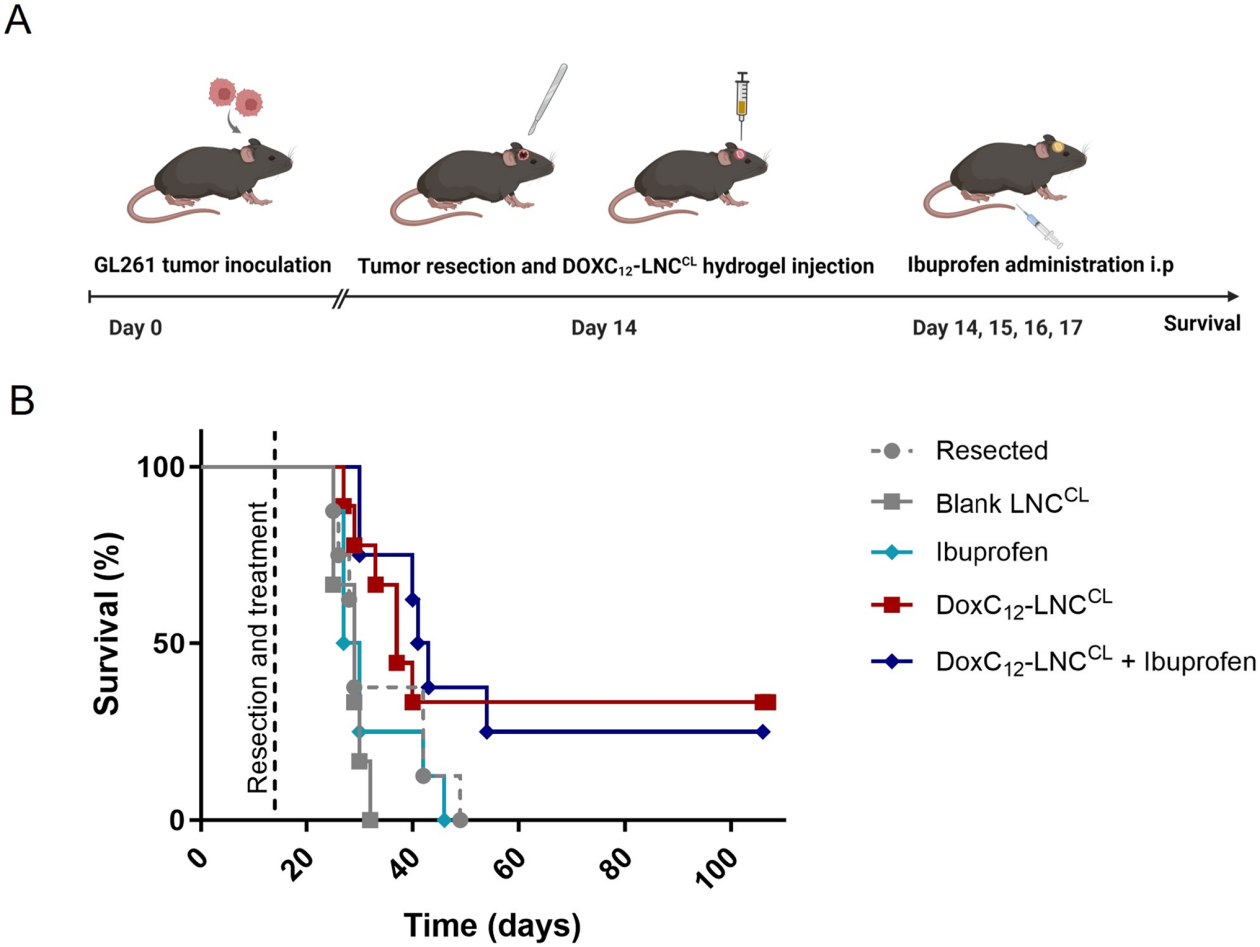
In vivo efficacy studies in a GL261 GBM-resected mouse model. **A** Schematic diagram of the timeline of orthotopic tumor inoculation, resection, and treatment administration. DOXC_12_ was locally administered at a dose of 5 mg/kg (5 μL of DOXC_12_-LNC^CL^ hydrogel, 20 mg/mL). Ibuprofen was systemically administered post-surgery and every 24 h for 3 days at a dose of 30 mg/kg. **B** The Kaplan–Meier survival curves of mice treated with different interventions

The anti-tumor efficacy of the DOXC_12_-LNC^CL^ hydrogel was then evaluated in a clinically relevant orthotopic GL261 GBM following tumor resection and treatment administration in the post-surgical cavity. The amount of DOXC_12_-LNC^CL^ to be administered was selected to 5 mg/kg considering the differences in administration techniques compared to the proof-of-concept study (CED *vs* resection cavity), the DOXC_12_-LNC^LC^ release profile, and technical constraints. To potentially increase the efficacy of the local treatment, we also combined it with the systemic administration of ibuprofen (Fig. 8A). The post-surgical administration of anti-inflammatory drugs has been shown to prevent the onset of recurrences in several tumor models including GBM [37, 69, 70]. The Kaplan–Meier survival analysis (Fig. 8B and Table 2) revealed that treatment with blank LNC^CL^ or ibuprofen alone did not significantly increase the median survival compared to the resected animals who did not receive any treatment. However, treatment with the DOXC_12_-LNC^CL^ hydrogel showed an improvement in median survival of the animals (37 days) compared to the resected untreated animals (29 days, not statistically significant) and the blank LNC^CL^-treated group (29 days, **p < 0.01). Remarkably, when the DOXC_12_-LNC^CL^ hydrogel was combined with repeated ibuprofen administration, there was a substantial increase in median survival (42 days) compared to the resected untreated animals (29 days; *p < 0.05) and the blank LNC^CL^-treated group (29 days; **p < 0.01). Both the DOXC_12_-LNC^CL^ and DOXC_12_-LNC^CL^ + ibuprofen groups presented several animals who responded as long-term survivors and lived without tumor relapse for more than 3 months.

**Table 2:** In vivo efficacy studies in a GL261 resected mouse model: median survival (days), numbers of long-term survivors in each group, and statistical analysis

Treatment	Median survival (days)	Long-term survivors	Log-rank test
Resected	29	0/8	
Blank LNC^CL^	29	0/6	ns (*vs* resected)
Ibuprofen	28.5	0/8	ns (*vs* resected) ns (*vs* blank LNC^CL^)
DOXC_12_-LNC^CL^	37	3/9	ns (*vs* resected) ** (*vs* blank LNC^CL^)
DOXC_12_-LNC^CL^ + Ibuprofen	42	2/8	* (*vs* resected) ** (*vs* blank LNC^CL^)

Log-rank (Mantel-Cox) test: survival curve comparison between groups

*p < 0.05, **p < 0.01, *n* 6–9 mice per group

The results confirm that LNC-based hydrogels are safe for administration in the post-surgical cavity. Indeed, as previously demonstrated [23, 25], the animals did not show any behavioral changes or body weight loss following treatment with blank LNC^CL^ or DOXC_12_-LNC^CL^ (data not shown). Moreover, we confirmed our hypothesis that DOXC_12_ formulated in an LNC-based hydrogel adapted for injection in the post-surgical cavity could increase the survival of the GBM-bearing mice and induce long-term survival. The significantly enhanced anti-cancer efficacy of DOXC_12_-LNC^CL^ in comparison to blank LNC^CL^, as observed in our in vivo experiments, aligns with the significance found in our ex vivo study using the brain slice model. Moreover, we showed that the combination of DOXC_12_-LNC^CL^ with ibuprofen led to a 5-day improvement in median survival when compared to monotherapy with DOXC_12_-LNC^CL^ hydrogel. Although not statistically significant, this difference shows that modulating the inflammatory response following surgery delays the onset of recurrences showing a promising approach for further experiments and combinatory approaches. Indeed, surgery induces neuroinflammation which might have a role in tumor relapse [36, 71, 72]. Even though the cross-talk between resident brain cells, immune cells, and residual GBM cells in the post-surgical microenvironment is still under investigation, it has been previously reported that modulating this environment can reduce tumor relapse [39, 69, 73]. Therefore, combining anti-inflammatory drugs with local chemotherapy can be a promising approach to induce long-term efficacy. Ibuprofen might promote the resolution of the local inflammation which might lead to a less protumorigenic microenvironment as well as acting synergistically with DOXC_12_ on residual tumor cells. Despite being a non-selective cyclooxygenase (COX) inhibitor, ibuprofen can reduce the overexpression of COX-2 by inhibiting arachidonic acid pathway metabolites (prostaglandin E2) and reducing STAT3 phosphorylation in microglial cells [70, 74]. The resolution of the proinflammatory tumor microenvironment could lead to a slowdown in the initiation of tumor regrowth by residual GBM cells, as they lose the supportive microenvironment typically characterized by active proliferative processes triggered by wound healing [71, 72]. Besides, ibuprofen can induce apoptosis of cancer cells through COX-independent mechanisms. These mechanisms include the downregulation of nuclear factor-kappa B (NF-kB) activity, modulation of pro- and anti-apoptotic protein levels, activation of extrinsic and intrinsic pathways of apoptosis, inhibition of proteasome function, cell cycle arrest, generation of stress responses, and activation of stress kinases [75, 76]. Thus, the observed survival benefit in animals treated with the combination of DOXC_12_-LNC^CL^ and ibuprofen may act through a diverse mechanism of action. Further exploration and preclinical studies will be necessary to fully understand the potential of this combined treatment in the management of GBM, also by modifying the treatment regimen. Overall, the combination of DOXC_12_-LNC^CL^ with ibuprofen represents a promising therapeutic approach for GBM treatment.

## Conclusion

Our study aimed to address the persistent challenge of GBM recurrence by exploring a novel approach: developing an injectable DOX-LNC-based hydrogel, DOXC_12_-LNC^CL^, to be administered into the resection cavity. We successfully developed this hydrogel without the need for an additional scaffold, and its mechanical properties were suitable for brain application. In vitro experiments demonstrated sustained drug release for over 1 month, indicating its potential as a long-term drug delivery system. DOXC_12_-LNC^CL^ exhibited potent anti-cancer efficacy in both 2D GBM cell cultures and 3D tumor spheroids on organotypic brain slices. Furthermore, in an orthotopic GL261 GBM preclinical model, the injection of DOXC_12_-LNC^CL^ into the tumor resection cavity resulted in increased survival rates and the presence of long-term survivors. Thus, this promising therapeutic strategy offers hope for delaying the onset of GBM recurrence. Further investigations will delve into the impact of DOXC_12_-LNC^CL^ in the GBM resection microenvironment to gain deeper insights into its mechanism of action.

## Data Availability

The datasets generated during and/or analyzed during the current study are available from the corresponding authors on reasonable request.
